# Factors Influencing the Adoption of IoT for E-Learning in Higher Educational Institutes in Developing Countries

**DOI:** 10.3389/fpsyg.2022.915596

**Published:** 2022-07-08

**Authors:** Syed Hamid Hussain Madni, Javed Ali, Hafiz Ali Husnain, Maidul Hasan Masum, Saad Mustafa, Junaid Shuja, Mohammed Maray, Samira Hosseini

**Affiliations:** ^1^School of Computing, Faculty of Engineering, Universiti Teknologi Malaysia, Skudai, Malaysia; ^2^College of Computing Informatics, Saudi Electronic University, Madina, Saudi Arabia; ^3^School of Civil Engineering, Faculty of Engineering, Universiti Teknologi Malaysia, Skudai, Malaysia; ^4^Department of Computer Science, COMSATS University Islamabad, Abbottabad Campus, Abbottabad, Pakistan; ^5^Department of Information System, College of Computer Science, King Khalid University, Abha, Saudi Arabia; ^6^Writing Lab, Institute for the Future of Education, Tecnologico de Monterrey, Monterrey, Mexico

**Keywords:** E-Learning, Internet of Things (IoT), adoption, influencing factors, higher education institutes (HEIs)

## Abstract

The internet of things (IoT) is an emerging paradigm of educational applications and innovative technology in the current era. While capabilities are increasing day by day, there are still many limitations and challenges to utilizing these technologies within E-Learning in higher educational institutes (HEIs). The IoT is well-implemented in the United States of America (USA), United Kingdom (UK), Japan, and China but not in developing countries, including Saudi Arabia, Malaysia, Pakistan, Bangladesh, etc. Few studies have investigated the adoption of IoT in E-Learning within developing countries. Therefore, this research aims to examine the factors influencing IoT adoption for E-Learning to be utilized in HEIs. Further, an adoption model is proposed for IoT-based E-Learning in the contexts of developing countries and provides recommendations for enhancing the IoT adoption for E-Learning in HEIs. The IoT-based E-Learning model categorizes these influencing factors into four groups: individual, organizational, environmental, and technological. Influencing factors are compared along with a detailed description in order to determine which factors should be prioritized for efficient IoT-based E-Learning in HEIs. We identify the privacy (27%), infrastructure readiness (24%), financial constraints (24%), ease of use (20%), support of faculty (18%), interaction (15%), attitude (14%), and network and data security (14%), as the significant E-Learning influencing factors on IoT adoption in HEIs. These findings from the researcher's perspective will show that the national culture has a significant role in the individual, organizational, technological, and environmental behavior toward using new technology in developing countries.

## Introduction

Higher educational institutes (HEls) are vast intelligent systems. This is due to the fact that the main components of current HEIs, educators, and educated learners are all information-complex holographic humans (Dhamdhere, 2015). Therefore, HEIs affect the knowledge, competence, and qualities of the educated person, bypassing information (inclusive of words and acts), remote conveyance, advocacy, counseling, and encompassing the educated self-learning. The presence of technology in education results in a vigorous collaborative self-directed model (Shaikh et al., 2019). Also, learner engagement in learning and content creation results from the influence of technology in education. The seven groups of technologies, tools, and approaches for managing revolution in education are consumer technologies, digital approaches, enabling technologies, technologies from the Internet, learning technologies, technologies related to social media and visualization technologies (Larrabee Sønderlund et al., 2019; Leal Filho et al., 2019).

Internet of things (IoT) provides massive opportunities for HEIs that bring together independent control and provision of better infrastructure robustness, scalability, and agility. The IoT allows humans and things to access from anywhere, anytime, and any place the link with anything and to any person without a specific path and service (Villa-Henriksen et al., 2020). Furthermore, the IoT spreads online teaching and learning to students and expands processes (Al-Emran et al., 2020). Moreover, the IoT saves costs and helps learners to take their classes at any time, within university premises, at home, or even on subways. Therefore, the IoT is expected to offer solutions that will alter teaching and learning activities (Ramlowat and Pattanayak, 2019).

The Internet of things is a new actor in learning environments. It plays a significant role in bringing interactivity, improved learning, and understanding between academic staff and learners *via* virtual and physical objects within the HEIs environment. Also, there is more focus on smart education and the use of IoT technology to bring improvement to learners in a class. Hence, with the increasing rate of utilizing online teaching by HEIs, assessment of the adoption of IoT is becoming dominant among academics and researchers (Kassab et al., 2020).

Internet of things supports the changes in the HEIs environment in education, including teaching, learning, management, experiment, training, school, campus building, etc. This creates a new opportunity where innovative learning options result from the change in concepts from ubiquitous computing and technologies. In developing countries, the Ministry of Higher Education Strategic Plan 2013–2020 requires the education sector to contribute, advance, and harmonize quality education, training, and research (Falqueto et al., 2020; Kenno et al., 2021). However, research shows that IoT adoption for E-Learning in HEIs is still in the early stages, with few papers focusing on it (Sani, 2019). The recent academic literature shows a gap in E-Learning for the adoption of IoT in developing countries. In the existing studies, most of the researchers focused only on technological factors. Hence the individual, organizational, and environmental factors also play an essential role in the adoption of new technology. Therefore, the researcher feels that there is a vital requirement for Saudi Arabian HEIs to adopt IoT to obtain improved opportunities.

**Figure 1:**
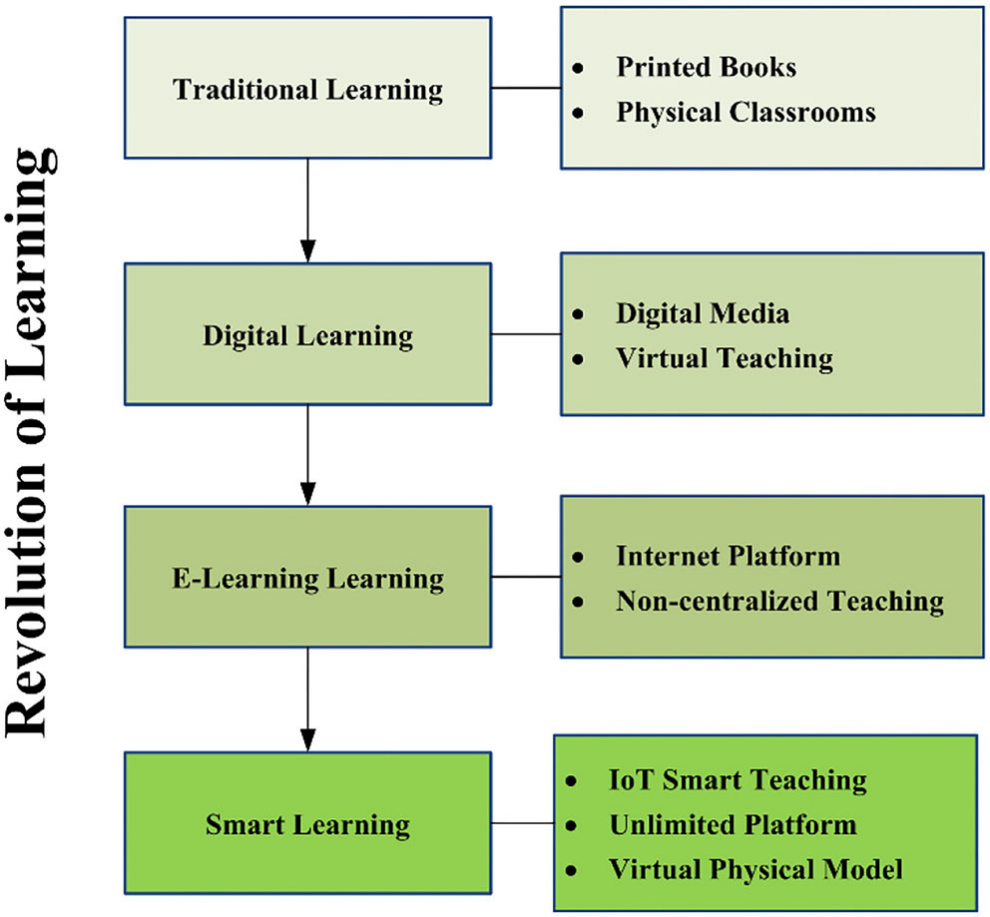
Revolution of learning.

The educational system has been transformed in most of the developing countries. At the same time, new specifications are required to establish teaching and learning methods for success within their setups and the boundaries that may entail. Technology is progressively essential in answering and allowing for innovative outcomes in terms of teaching and learning, such as the inverted classroom, massive open online courses (MOOCs), and smart learning. Till now, the revolution of learning is divided into four groups including traditional, digital, E-Learning, and smart learning, as shown in Figure 1 (Verma and Singh, 2021; Verma et al., 2021). In traditional learning, printed books are used, students went to school with those books, and teachers used to teach them on the blackboards or whiteboards. This learning method was physical, such as face-to-face classes (Zhong et al., 2016). Then digital learning brought considerable changes in the road of education, and it brought virtual learning. Students were able to learn on the Internet from different sources (Sousa et al., 2017).

Currently, we are living in the era of Smart learning. In simple words, Smart learning is the combination of E-Learning and IoT, or it is also known as IoT based E-Learning, as shown in Equation (1). In IoT based E-Learning, students learn from their educational institution or home with various IoT sources online. Similarly, instructors give the lectures in the form of different videos, meeting websites, and apps, while arranging the test also online by using the IoT or smart devices. Students feel the essence of physical classes in these virtual meetings or online courses (Kumar Basak et al., 2018). Obviously, Smart Learning or IoT based E-Learning effects enhance the students' performance in terms of achievement, knowledge, learning, and results (Saiyeda, 2021; Djeki et al., 2022).


(1) $$\begin{array}{l} IoT+E-Learning=Smart\;Learning \end{array}$$

Everything is digitalized in the modern era so the educational system has yet to incorporate the latest IoT technology. The education system has progressed through several stages of modernization from traditional learning to smart learning with E-Learning. Now, it is high time for the adoption of IoT in E-Learning in HEIs. Students can study effectively in IoT based E-Learning and the instructors can also easily teach them, just as they would enjoy in traditional schooling. They will have easy access to all course-related technologies, as well as a user-friendly interface *via* which they will be able to complete their test/quiz/exam online and have it graded automatically by the system. Their attendance will also be recorded by the system. Most significantly, after the COVID pandemic, IoT based E-Learning can provide an adequate education system for future generations. For this purpose, we examine, analyze, and compare the several existing studies relevant to the adoption of IoT based E-Learning. Also, it highlights the facilities required and identifies the influencing factors that must be addressed to create a successful IoT based E-Learning system in HEIs. Our contributions to this research are as follows:

- We review the existing studies on IoT based E-Learning and summarize them to emphasize the concerns raised.
- We examine the adoption of IoT based E-Learning in HEIs in-depth, comparing the system's functioning to create a smooth and effective system.
- Based on the existing studies, we highlight the influencing factors for the adoption of IoT for E-Learning in HEIs.
- We propose the IoT based E-Learning Model in terms of the adoption of new technology in the education system.
- Further, we categorize these influencing factors into four groups: individual, organizational, environmental, and technological for adopting IoT for E-Learning in HEIs.
- Finally, we present the comparative analysis of these influencing factors with a detailed description in order to determine which factors should be prioritized for efficient IoT based E-Learning in HEIs.

However, reviews, evaluations, and comparisons of existing relevant articles assist in understanding the general concept and identifying the aspects of IoT adoption that need more attention, improvement, and generate suggestions for future research. This research will help future researchers understand the existing state of education and the requirements for adopting IoT for E-Learning in HEIs. The remaining sections of this study are structured as follows:

Section Related Works focuses on evaluating and examining the relevant related surveys and reviews the adoption of new technologies for E-Learning in HEIs. Section Problem Statement discusses the problem formulation that defines the drawbacks of the present education methods and the need to switch to IoT-based E-Learning systems in HEIs. The research methodology of preparing this study, including the inclusion and exclusion of existing studies, is addressed in section Methodology. Section Existing IoT Based E-Learning System in HEIs explains the entire system used for IoT-based E-Learning and how it works and is addressed by existing researchers. One of the most important portions of this research is section Categorization of E-Learning Influencing Factors of IoT Adoption in HEIs, the proposed IoT-based E-Learning model, which is the categorization of E-Learning influencing factors of IoT adoption in HEIs. Section Comparative Analysis of the Influencing Factors for the Adoption of IoT for E-Learning in HEIs presents the comparative analysis of influencing factors, studies and brings out the factors that need more attention. Furthermore, the study ends in section Conclusion and Recommendations by presenting the conclusion and some recommendations for the improvement of IoT based E-Learning in HEIs.

## Related Works

In this section, we study the existing relevant reviews and survey studies for the adoption of new technologies in education systems. Influencing factors regarding the students are also explored while addressing the open issues and challenges for further research. Hence, Kamarudin et al. (2019) propose the mobile application framework for education support with the help of IoT in terms of the Malaysian context. Similarly, Wangoo and Reddy (2020) review the various graphical user interfaces (GUIs) in mobile apps for the development of educational applications. Also, a smart learning framework is proposed based on IoT that enables the GUI interface for smart educational systems. The smart learning framework is used for administrative activities, monitoring, tracking, learning, and reporting in educational institutions. In the same way, Sezer et al. (2017) introduce context-aware computing to highlight the growth of IoT related to learning and big data and various research fields regarding management and analytics. For this purpose, machine learning algorithms based on supervised learning and unsupervised learning are reviewed in detail. The open issues and challenges of IoT are also identified for further research enhancement.

Ejaz et al. (2020) present IoT research, including the integration of learning techniques from an optimization viewpoint, with optimization targets classified as maximization, reduction, and associated applications. It also covers learning algorithms for key performance indicators (KPIs) used to evaluate the performance of IoT systems. The topic of learning techniques is explored and demonstrated how these methods might help IoT apps perform better. Finally, potential research prospects for applying learning methods to improve IoT systems are also discussed. Further, Rico-Bautista et al. (2019) observe the growing perception of smart universities related to the IoT. The goal is to identify the hypothesis, justification, problem situation, and conceptual framework published in the scientific literature about the terms and characteristics of smart universities, from which the role of the IoTs is emphasized as an essential component in the implementation and conception of projects and initiatives that affect the successful development of educational organizations. Similarly, Kaizer et al. (2020) conduct a descriptive and bibliometric evaluation of the literature on the instruction planning for e-learning training in the workplace. The latest techniques and experiences are used as a model for professionals preparing e-learning training in the workplace are also identified. The elements of the training planning stage, regardless of the delivery method, are identified. It is considered a set of activities that make up the training and development system or instructional technology (such as face-to-face and e-learning).

Innovative applications of IoT, campus model, and implementation of IoT reviewed in university campuses are based on IoT entrance systems, flipped classrooms, orangery heating systems, and students' feedbacks. Moreover, the impact of IoT flipped classrooms and traditional education according to students' feedbacks is judged. Also, the benefits of IoT applications are highlighted, including smart building, smart learning, sustainability, technology awareness, and waste and water management (Zhamanov et al., 2017). In the same way, Lei et al. (2017) propose the teaching of IoT with the help of flipped classrooms for enhancing learning efficiency. Fourteen students are enrolled in the IoT development course which allows for flexibility in learning and enhances learning style and needs while allowing instructors to make quality use by conducting class activities, individual interaction, and coaching. In conclusion, the IoT development course helps increase the impact of teaching and learning. However, Qasem et al. (2019) present the Systematic Literature Review (SLR) for evaluating the existing studies on cloud computing adoption in HEIs. The SLR is based on research background, comprehensible taxonomy, and future directions. The taxonomy is characterized by the basics of barriers, motivation, individual models, organizational models, and adoption of cloud computing at various levels in HLIs that help in further research in terms of more scientific and technological contexts. Furthermore, Popchev et al. (2019) propose the conceptual model of E-Learning in terms of IoT adoption. For this purpose, Big Data analytics are identified based on the diagnostic, descriptive, predictive, and prescriptive manners. Existing techniques used by researchers in big data analysis are also identified.

Internet of things is used to track and trace a learner's attendance in the education department for the primary purpose. Chweya and Ibrahim (2021) present IoT implementations, the application areas, the models and techniques that are utilized, and their outcomes at educational institutions. The findings of the study specify that IoT implementation might aid in the resolution of various difficulties in educational institutions, such as equity and learning quality. The findings also serve as a foundation for further research on the practical implementation of IoT in educational institutions. Moreover, Gul et al. (2017) examine the significance and uses of the IoTs in the domain of education. Security, privacy, availability, cost, reliable Wi-Fi connection, mobility, dependability, performance, interoperability, scalability, trust, and management are significant challenges for IoT in education that are enlisted and pointed out.

The digital transformation reveals the new perceptions to change the way of thinking, learning, and implementing things in real life and, more specifically, in the further education systems. Similarly, Zahedi and Dehghan (2019) discuss the IoT adoption-based E-Learning in several aspects, including the benefits, importance, necessity, current challenges, possible solutions, and future directions. In the same way, Pervez et al. (2018) describe the aspects of IoT productivity for the trainers and learners, which show the various learning trends and success ratios. Further, Khan and Salah (2020) propose the study of a cloud-based survey to enhance the students' skills in the educational environment. For this purpose, we present the cloud usage taxonomy for E-Learning and analyze models and frameworks of existing studies. Future work is also mentioned for adopting cloud technology in E-Learning with possible solutions.

Muzaffar et al. (2021) conduct a systematic review of online exam solutions for E-Learning, including the tools, techniques, and global adoption. Important attributes with respect to the online examination system, including the hardware requirements, network infrastructure, training requirements, and implementation complexity, are also identified. Furthermore, substantial challenges are also highlighted for online exams, such as integrity, reliability, and security. In the same way, Letting and Mwikya (2020) perform a qualitative review on IoT and the quality of HEIs in Kenya. The HEIs need to reduce the latency time due to the high demand for content in instructional technologies. The modern electronic classroom must be equipped with a lecture capture system and web streaming tools, which provide an opportunity for the students to access the lectures at any time.

Matthew et al. (2021) review the contemporary development of IoT and cloud computing in E-Learning systems and establish the prospect for continued educational investment. Manageability issues are also highlighted in E-Learning to explore performance. Moreover, Rahmani et al. (2021) examine the E-Learning development based on blockchain and IoT with the help of existing and future conditions that enable E-Learning applications in terms of architectural designs. Furthermore, Djeki et al. (2022) present the bibliometric analysis of E-Learning from 2015 to 2020 to highlight the collaborations, identify the most influential research entity, and know the issues and gaps in the field.

Recently existing studies focus on the E-Learning technologies, developments, strategies, and methodologies based on IoT, blockchain, and cloud computing. Hence, this research identifies the factors influencing the adoption of IoT for E-Learning in higher educational institutes in developing countries.

## Problem Statement

There is intense worldwide pressure on creating and maintaining a balance between the demands of quality, equity, and funding in enrolments in the face of fast expansion. However, research agrees that large classes affect the quality of education in relation to the environment for learning. Hence, higher education is regularly searching for ways to increase the number of learners in a cost-effective way and transfer knowledge in high quality. In addressing the above, the IoT has been recognized as a tool to solve the occurrences of the related issues (Ding et al., 2020).

In comparison with the traditional E-Learning systems, the IoT provides a novel electronic teaching and learning platform with many distant learning objects (Abbasy and Quesada, 2017). The IoT has been long developed in developed countries, such as the United States of America (USA), the United Kingdom (UK), Japan, and China. However, this study noted that developing countries, including Saudi Arabia, Malaysia, Pakistan, Bangladesh, etc., lack IoT in education systems. Clearly, higher learning systems have the least experienced changes brought by the incorporation of technology and virtual teaching/learning methods, E-Learning, M-Learning, and U-Learning (ubiquitous) (Moreira et al., 2017; Razzaque and Hamdan, 2020b).

The recent academic literature shows a gap in IoT adoption in HEIs for E-Learning in developing countries. According to this research study, there is limited research about the adoption of IoT for E-Learning in higher educational institutions in developing nations. However, research shows that IoT implementation in higher learning is still in the early stages, with few research articles focused on it. Besides, there is a gap in evaluating the effect of individuals, organizational, technological, and environmental contexts on adopting IoT for E-Learning in HEIs in both developed and developing nations.

“*How to find the influencing factors of Internet of Things (IoT) Adoption in Higher Educational Institutions (HEIs) for E-Learning?”*

## Methodology

This section presents the research steps followed to perform this study. According to Moher et al. (2015), a comprehensive review of existing studies has been done in the research domain of IoT for E-Learning in HEIs to find the adoption factors. The research methodology requires the relevant papers from various databases, including the Web of Science, Taylor & Francis, Springer, Scopus, Science Direct, Google Scholar, IEEE Explore, and ACM Digital Library. This research study is based on the existing studies from 2016 to 2021. We define the search key strings that give us the sufficient amount of most suitable related research studies: (“E-Learning” ^*^ “Internet of Things” + “IoT” + “Higher Educational Institutes” + “HEIs”) and (“E-Learning” AND “Internet of Things” OR “IoT” OR “Higher Educational Institutes” OR “HEIs”).

In the screening process, duplicate studies are removed at the first stage. After that, exclusion criteria have been applied based on the title, abstract, and body of the research article. The inclusion and exclusion criteria have also been applied on the basis of the study focusing on adopting new technology for E-Learning, written in English, and published in well-recognized journals and conferences. Moreover, content analysis has been performed to categorize the influencing factors, and a comprehensive list of E-Learning factors for IoT adoption in HEIs is compiled.

## Existing IoT Based E-Learning System in HEIs

This section explains the entire used system for IoT-based E-Learning and how it works and is addressed by existing researchers.

In two universities in Saudi Arabia, a quantitative analysis is conducted with the help of 527 students. This analysis is shown that 96.7% of students are keen on adopting IoT technologies in the higher education system. In this analysis, two main factors, perceived usefulness and ease of use, are examined by using Technology Acceptance Model 3 (TAM3). Apart from other factors, the trust factor is also included in considering the importance of adopting IoT technologies in E-Learning based on systems of higher education systems (Almazroi et al., 2016). Similarly, Ngampornchai and Adams (2016) study the undergraduate student perceptions of E-Learning and consciousness of innovative technologies in the rural area of Thailand for identifying the specific features that affect E-Learning adoption. The results of the survey show specific visions that may lead to E-Learning strategies and effective policies. In the same way, El-Masri and Tarhini (2017) examine the CSFs that allow for the web-based learning system in developing countries by using the Unified Theory of Acceptance and Use of Technology 2 (UTAUT2). The differences or similarities are explored among the American and Qatari students during the adoption of the E-Learning system.

Cloud computing-based E-Learning methods provide cutting age communication and information exchange methods in terms of assignments, quizzes, online exams, etc. Naveed et al. (2017) review the several critical success factors (CSFs) from the existing studies and identify the most significant CSFs of E-Learning in the HEIs of Saudi Arabia. These CSFs are classified into five dimensions, including the individual (instructor and student), organizational (institutional management services, design, and contents), and technological (technical and system) contexts. Further, Naveed et al. (2019) analyze the CSFs based on four dimensions: cloud-based E-Learning imperatives, cloud service resilience, organizational readiness, and technological maturity. Moreover, Alhabeeb and Rowley (2018) investigate and analyze the CSFs in HEIs from the perspectives of the students and academic staff and categorized them into two groups.

E-Learning always supports improving and enhancing the literacy rate of rural and urban users. A deep analysis of E-Learning factors provides policy makers with guidelines for enhancing and promoting the E-Learning system in HELs. Kanwal and Rehman (2017) highlight the external factors that consist of computer anxiety, use of the Internet, and self-efficiency, which impact the users' adoption of E-Learning systems. Similarly, Jović et al. (2017) examine the factors that influence students' attitudes toward E-Learning. The technology acceptance model (TAM) theory is used to find the relationship between TAM variables and attitudes toward E-Learning with the help of using principal component analysis (PCA).

Rahardjo (2018) identifies the relationship between E-Learning technology and readiness for adoption in terms of online classes that increase student enrollment rates. Student adoption or acceptance level used in the learning process is based on the technology through the online classes. In the same way, Muhammad et al. (2020) classify the effect of three dimensions, including the E-Learning environment provided to students (EES), awareness about academic integrity (AAI), and E-Learning guiding principles (EGPs) provided by the institutions with the help of AHP and Delphi strategies. Moreover, Al-Araibi et al. (2019b) propose a model for the technological feature of E-Learning readiness in HEIs, based on seven technological factors: connectivity, cloud computing/datacenters, the flexibility of the system, hardware software, security, and technical skills/support. This model shows that all seven factors significantly impact the readiness factors of E-Learning in HEIs except cloud computing.

To identify the effect of the CSF on E-Learning acceptance, Salloum et al. (2019) propose a model for investigating the influence of innovativeness, knowledge sharing, quality, and trust on E-Learning acceptance. It is observed that the E-Learning systems are not efficient without achieving the quality of the system. So, quality and knowledge sharing are considered the main factors that affect the E-Learning systems in terms of efficiency. Further, Eze et al. (2018) examine the nature of adoption and utilization of E-Learning facilities by lecturers in private Nigerian HLIs. The critical factors and issues that influence E-Learning adoption are also identified, including the facilities, preferences, and ease of use. Moreover, Al-Araibi et al. (2019a) evaluate the technological factors of E-Learning readiness by implementing of Delphi technique and designing the basic model for HLIs. The model is used to help the HLIs for identifying and understanding the technological aspects. These aspects must be considered for applying the E-Learning project by evaluating the readiness.

The IoTs are the next technological evolution that helps improve the various elements of education, such as E-Learning and U-learning. Moreover, the acceptance of IoT applications gives rise to worldwide innovative possibilities and challenges for E-Learning. Performance, motivation, financial constraints, and social impact are the variables at the individual level of E-Learning adoption. The influence of the usage of IoTs on students' academic performance is investigated, and the attitudes of Damietta university students about the consequences of IoT technology are determined (Amasha et al., 2020). Similarly, Mehta et al. (2019) investigate the individual level influence values for E-Learning adoption. Also, the values-enhanced technology adoption (VETA) model is developed and evaluated as a student perception of E-Learning adoption in the UK and Gambia. The UTAUT2 model is used to determine the influence values of the VETA model.

In the same way, Pérez et al. (2021) examine the attitudes and perceptions of instructors regarding the change in the smart learning space (SLS) environment in the various school levels (such as primary, secondary, and high) in Catalonia (Spain). The results show that instructors had less perception of adopting the SLS that identifies the need for educational reflection about change. Similarly, Shaikh H. et al. (2019) propose a network analysis technique for observing users' behavior in e-learning adoption of various IoT projects, such as multimedia usage in the classroom and digital library access. The significant benefits of IoT adoption in e-learning include performance expectancy, social influence, effort expectancy, social influence, hedonic influence, and positive behavioral intention. The results show that acceptance and usage of IoT are necessary for HEIs in Pakistan. Further, Shinghal et al. (2020) present IoT-based modified hybrid blended learning model for HEIs. The protocol of the proposed modified hybrid-blended learning model automatically detects the whole system and performs the tasks, such as delivering messages to students, grouping students, and implementing the plan. The results show that the hybrid-blended learning model is beneficial for both students and instructors to share knowledge and work in a blended learning environment online and face-to-face. Moreover, Kuliya and Usman (2021) analyze the perceptions of e-learning among the undergraduate students and academic staff of HEIs in north-eastern Nigeria. The results show that IoT adoption is considered a significant E-Learning component in developing countries. For successful implementation of E-Learning attributes, such as government support, budget allocation, and implementation strategies are also discussed.

Table 1 elaborates on the existing E-Learning studies that influence the adoption of new technologies for E-Learning in HEIs. Mostly, researchers try to find the critical success factors for the adoption or readiness of new technologies based on cloud computing and IoT. TAM, UTAUT, and UTAUT2 models are used to adopt the readiness of new technology for E-Learning. Both quantitative and qualitative methods are used with the help of most online surveys and interviews in developing countries, including Egypt, Gambia, India, Indonesia, Iran, Korea, Nigeria, Pakistan, Qatar, Saudi Arabia, Spain, and Thailand.

**Table 1:** Existing E-Learning studies that influence the adoption of new technologies for E-Learning in HEIs.

**References**	**Objectives**	**Theories/** **frameworks**	**Achievements**	**Methods**	**Data collection method**	**Countries**
Almazroi et al. (2016)	Use of cloud services for E-Learning activities and outcomes	TAM3	Improved the students' adoption	Quantitative	Survey questionnaire distributed personally	Saudi Arabia
Ngampornchai and Adams (2016)	Awareness of E-Learning	UTAUT	Improve the innovation adoption	Quantitative	Survey questionnaire distributed personally	Thailand
Kanwal and Rehman (2017)	Factors affecting E-Learning adoption	TAM	Implement successful E-Learning systems	Quantitative	Survey questionnaire distributed personally	Pakistan
Jović et al. (2017)	Attitude toward E-Learning	TAM	Identify the main factors	Quantitative	Survey questionnaire distributed personally	Serbia
El-Masri and Tarhini (2017)	Factors affecting E-Learning adoption	UTAUT2	Adoption of web-based learning systems	Quantitative	Survey questionnaire distributed personally	Qatar and USA
Naveed et al. (2017)	Identify the critical successful factors of E-Learning	Not mentioned	Identify the E-Learning variables and their effect on the use of E-Learning	Mix method	Survey and recent literature review	Saudi Arabia
Naveed et al. (2019)	Identify the critical successful factors of cloud based E-Learning	Multi-criteria decision making (MCDM)	Controlling and improving the teaching learning system	Analytic Hierarchy Process (AHP), Group Decision Making (GDM), and Fuzzy AHP (FAHP)	Not specified	Not Mentioned
Alhabeeb and Rowley (2018)	Identify the critical successful factors of E-Learning	Not mentioned	Identify the E-Learning variables related to staff and students	Quantitative	Survey questionnaire distributed personally	Saudi Arabia
Hamidi and Chavoshi (2018)	Adoption of mobile learning	TAM	Mobile learning is helpful for to use of resources.	Quantitative	Survey questionnaire distributed personally	Iran
Rahardjo (2018)	E-Learning technology and readiness adoption	TAM	Identify the relationship between E-Learning technology and readiness adoption	Quantitative	Survey questionnaire distributed online	Indonesia
Muhammad et al. (2020)	Identify the factors of violating academic integrity	Not mention	Academic integrity into E-Learning	Delphi	SPSS	Saudi Arabia
Al-Araibi et al. (2019b)	Identify the critical successful factors of E-Learning readiness	Not mention	Develop a model to identify the readiness factors for E-Learning	Quantitative	Survey questionnaire distributed online	Malaysia
Eze et al. (2018)	Adoption and utilization of E-Learning facilities	Not mention	Identify the factors of E-Learning	Qualitative	interviews	Nigeria
Salloum et al. (2019)	Identify the factors of accepting E-Learning	SEM	Quality and knowledge sharing is a successful key factor	Quantitative	Survey questionnaire distributed online	UAE
Al-Araibi et al. (2019a)	Investigate the technological factors of E-Learning readiness	Delphi technique	Develop a model for Technological aspects of E-Learning Readiness	Quantitative	Questionnaire distribution	Not Mentioned
Hasani et al. (2020)	Identify the readiness factors of E-Learning	SEM	Identify the factors of students' perceived readiness	Quantitative	Survey questionnaire distributed online	Indonesia
Amasha et al. (2020)	Identify the future use of IoT in E-Learning	Not mentioned	Present the significance of IoT in E-Learning	Quantitative	Survey questionnaire distributed personally	Egypt
Mehta et al. (2019)	Identify the individual level of E-Learning readiness	UTAUT2	Predict the behavioral intention	Quantitative	Survey questionnaire distributed online	Gambia and United Kingdom
Kim et al. (2019)	Digital readiness of E-Learning	PLS-SEM	Enhance the effective adoption of E-Learning	Quantitative	Survey questionnaire distributed online	Korea
Pérez et al. (2021)	Smart learning space	Not mentioned	Improving teaching and innovation	Quantitative	Survey questionnaire distributed online	Catalonia (Spain)
Shaikh H. et al. (2019)	Acceptance of IoT in HEIs	UTAUT2	Discover the benefits of IoT in E-Learning	Propose a model	No need	Pakistan
Shinghal et al. (2020)	Acceptance of IoT in HEIs	Blended learning model	Highlights the benefits of IoT	Propose a model	Raspberry pi microcontroller-based system	India
Kuliya and Usman (2021)	Perception of E-Learning	TAM	Enhance the effective adoption of E-Learning	Quantitative	Survey questionnaire distributed online	Nigeria

*MCDM, Multi-Criteria Decision Making; PLS-SEM, Partial Least Squares Structural Equation Modeling; SEM, Structural Equation Modeling; TAM, Technology Adoption Model; UTAUT, Unified Theory of Acceptance and Use of Technology*.

## Categorization of E-Learning Influencing Factors of IoT Adoption in HEIs

**Figure 2:**
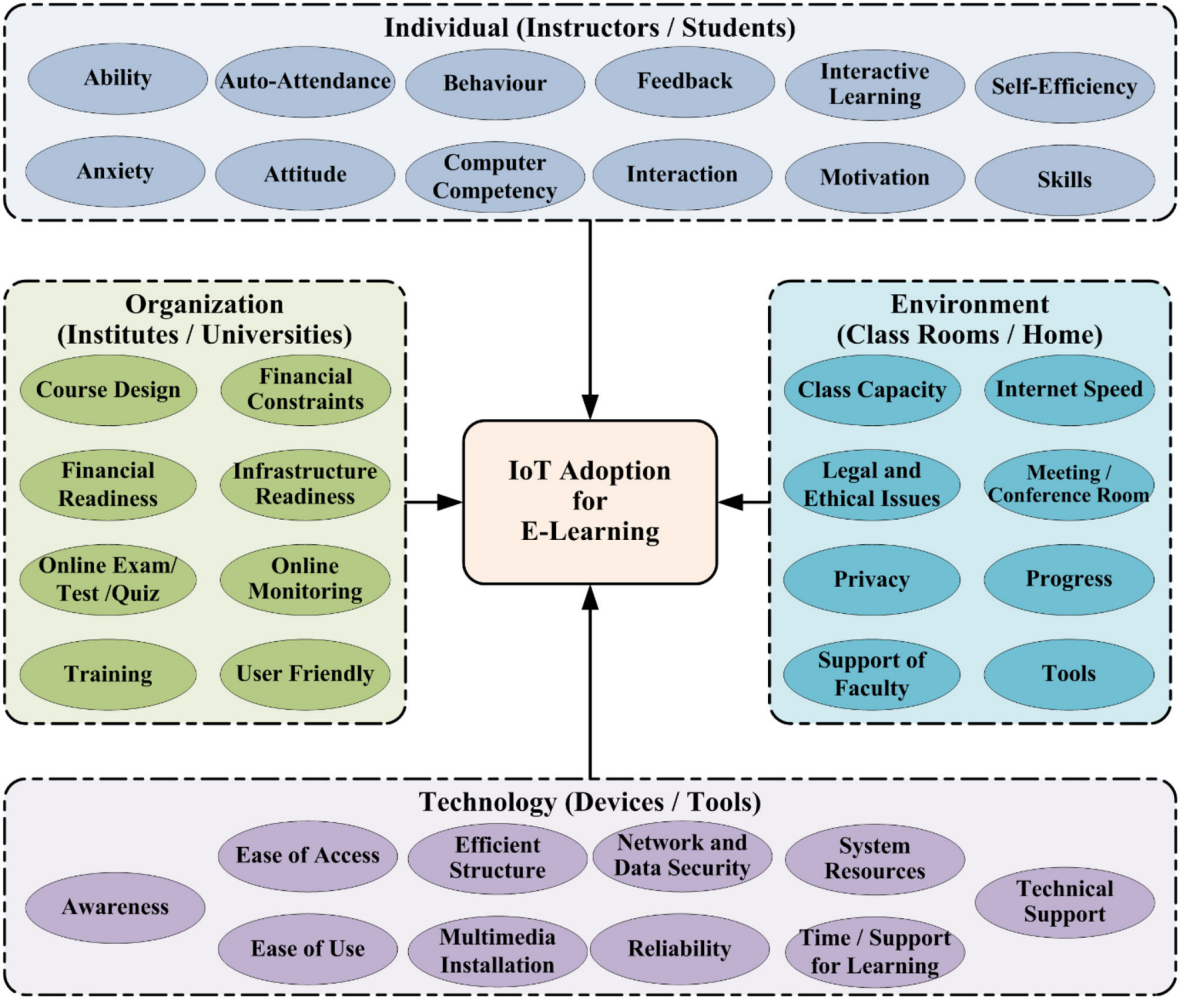
E-Learning-based IoT adoption model for higher educational institutes.

Adoption of the IoT is vital for effective E-Learning. Individuals and organizations must work in a technological environment where specific processes must be followed in order for learning to be successful and effective. For this purpose, E-Learning influencing factors of the IoT adoption model in HEIs are classified into four groups: individuals, organizational, technological, and environmental, as shown in Figure 2. Individuals are based on instructors and students, organizations depend on institutes and universities, technology is based on devices and tools, and the environment depends on classrooms and homes. Furthermore, details are described in subsections as given below:

### Individual Factors

Individuals consist of instructors or students who must possess specific IoT-based E-Learning skills to function successfully. Individuals must have computing abilities, and it is recommended that they remain self-motivated by realizing and learning a lot from IoT based E-Learning approaches. They must be cautious in their actions, attitudes, and everyone must maintain a high level of morality. In order to learn effectively, students and instructors must engage in interactive learning sessions. Instructors may obtain feedback from students on how the learning process is going on and if they understand the learning material correctly or need clarification. Instructors can also automatically use the IoT-based E-Learning sign-in record to automatically save attendance (Agrawal et al., 2020). Individual factors are described in subsections as follows:

#### Ability

Ability means having the power or source for efficiently accomplishing specific tasks. To implement E-Learning, instructors must have a high level of educational ability, such as creativity, adaptability, problem solving, and critical thinking. E-Learning flourishes better teamwork between students and instructors by using IoT and smart devices. The ability of students should be encouraged to deal with innovations and learn in high-tech environments (Supriyatno et al., 2020).

#### Anxiety

Anxiety is an emotion comprising worry, fear, and tension about whether someone is able to do tasks or not. From a student's perspective, anxiety occurs when the E-Learning interface is not easy to understand and provides a learning environment. E-Learning is highly reliant on instructors' and students' evolution and their willingness to shift from old teaching and learning techniques toward high-tech learning environments. When it comes to investing in IoT-based E-Learning systems, there are financial concerns among students and instructors. The equipment and technology are costly in terms of IoT and smart devices that must be purchased to execute E-Learning properly (Saadé et al., 2017; Zahedi and Dehghan, 2019).

#### Auto Attendance Mechanism

It is comfortable and time saving for students and instructors to use the automatic attendance system simply by login onto the digital E-Learning platform by using smart devices. Moreover, students who are unable to attend class due to illness or other reasons can access lectures *via* a smart e-learning system at any time. The Smart-Class keeps track of students' attendance and provides a unique learning platform that promotes cyber engagement and collaboration. Also, IoT-based E-Learning provides facilities and leniencies to the instructors to upload course materials and manages the class work anytime and at any place without the restriction of on-campus physical attendance (Ghonaim et al., 2018; Shah and Barkas, 2018).

#### Attitude

Attitude is a combination of emotions toward objectives, events, and assumptions. Instructors use e-learning to assist teaching, feel confident in giving online lectures, and promote distance learning positively. When students' attitudes toward e-learning become more optimistic, there is a better interactive intention to use IoT or smart devices. Students expect the instructors to assist and guide them in a positive way by utilizing the high-tech for IoT-based E-Learning environments (Nyagorme, 2018).

#### Behavior

Behavior is a way to act or accomplish something in a certain manner. Behavior is linked to every aspect of life, including the adoption of IoT-based E-Learning. E-Learning courses need a clear goal, internal motivation, synchronous feedback, and learner freedom. However, student learning behaviors directly influence every student on their roster in terms of their progress or achievements. Furthermore, specific instructional plans might be crushed entirely when a student's behavior interrupts the entire class. Instructors must also assist students in completing one or more online learning experiences in terms of behavior (Almajali, 2020; Mailizar et al., 2021).

#### Computer Competency

Computer competency means having a grip or knowledge of computer-related tools, technologies, and devices. Computer competency is necessary for students and instructors to implement IoT-based E-Learning. Instructors usually have expertise in face-to-face training and instructions. Through the E-Learning platform, instructors can easily produce lectures that can be posted online and teach the students with the help of smart IoT apps and devices from any time and any place. Students can access lectures by using web browsers while at home just by installing and using the specific IoT apps for E-Learning (Raman et al., 2019).

#### Feedback

The information or opinion regarding something to improve the quality is known as feedback. IoT- based E-Learning platform improves the teaching and learning experiences of HEIs students and instructors. Fast feedback from the instructor is possible within an IoT-based E-Learning environment. While teaching, instructors execute a variety of tasks like providing a structure for the course materials, providing feedback on successes, stimulating students' motivation to process, and assisting them in participating in learning activities (Tancock et al., 2018).

#### Interaction

The act of involvement in some concerns, activities, and matters is known as interaction. E-Learning allows for instant interaction between students and instructors just by signing into the online platform or contacting with her/him *via* e-mail, chat, and forum. High interaction between students and instructors promotes the formation of shared course materials and activities to refine the learning process effectively and improve the overall student performance. Students' sense of connection, influence, and stickiness with the IoT-based E-Learning platform is greatly enhanced by student–instructor interaction and student–student interaction in individual and group learning activities (Kokoç and Altun, 2021).

#### Interactive Learning

Enjoyable and interesting interactive learning environments enhance learners' self-regulation toward e-learning. Learners in an E-Learning system communicate interpersonally with the help of IoT and smart devices rather than face-to-face contact. For interactive learning, instructors provide interactive learning platforms, such as videos, online individual and group activities, or assignments and projects to enhance students' performance in terms of learning and learner satisfaction for IoT- based E-Learning environments (Nariman, 2020).

#### Motivation

Motivation is an act or process of giving someone the enthusiasm to do things efficiently and effectively. E-Learning encourages students and instructors to participate more actively on IoT platforms. The most challenging responsibility of instructors is to keep students motivated and curious about relevant subjects of the specific course in terms of learning. Enthusiastic, smart attitude toward students, innovative teaching approaches, and effective handling of E-Learning elements are the important attributes of the instructions to enhance students' motivation (Fernando et al., 2019).

#### Self-Efficiency

Self-efficiency refers to someone's confidence or belief to accomplish specific tasks competently. E-Learning enhances self-efficiency by allowing students and instructors to keep the information up to date, maintain course material in a timely and effective manner, and enables students to view in-class activities and listen to instructors as many times as they need (Nyagorme, 2018). E-Learning effectiveness is impacted by using IoT or smart devices, multimedia instructions, interactive learning activities, and the quality of the teaching and learning abilities.

#### Skills

The ability to perform or execute a task proficiently is known as skill. IoT-based E-Learning allows students to set their own pace of learning and the use of learning strategies in assignments, and gain knowledge and skills in the subject matter. By using IoT-based E-Learning, instructors enhance the skills of the students in terms of gaining knowledge, communication, and cooperation skills, the progress of self-regulated, and understanding of the learning skills for professional practice (Fernando et al., 2019).

### Organizational Factors

Organizations consist of institutes and universities. On the other hand, organizations must keep a few things in mind, such as designing the course before publishing it and making the website user-friendly. They should also examine if they are financially ready to publish a course and can meet all the requirements in the future before doing so. Also, they should make sure that the website is infrastructure-ready so that an online exam/test/quiz can be taken and monitored if all goes as planned (Ahmad et al., 2021). Organizational factors are described in the following subsections.

#### Course Design

Academic quality, course assessment, and methodology in e-learning can enhance students' performance by using IoT or smart devices. The learning content is essential in encouraging motivation in the students' performance. There should be official guidelines by HEIs in formulating course strategies by considering IoT-based E-Learning. Teaching staff should be provided with the design, operational plans, and development support when engaging in course design for IoT-based E-Learning (Almaiah and Alyoussef, 2019).

#### Financial Constraints

Education board provides E-Learning solutions to emphasize complete customized and adaptable packages of IoT or the cost of smart devices for those HEIs use to meet their specific needs and objectives by using E-Learning technology (Agabi, 2019; Nie et al., 2020). The cost of IoT smart devices or technologies is higher compared to the conventional learning system. Moreover, E-Learning also requires high development and maintenance costs due to the adoption of high-tech. HEIs allocate the budget for the implementation of e-learning technologies and applications is ~ <25% of the total budget.

#### Financial Readiness

Financial readiness determines whether the organization (Institute or University) is financially ready to pursue its objective. For example, IoT-based E-Learning necessitates a large number of tools and software and specialists to handle, all of which demand a large sum of money. The organization must consider these factors before deciding whether to implement an IoT-based E-Learning system. They must figure out how much it will cost in a month or a year. The organization must plan appropriately to make the most effective use of its funds. They must preserve all records, save money for future requirements, and be alerted to avoid needless spending while they spend (Ahmad et al., 2021; Purba et al., 2021).

#### Infrastructure Readiness

Infrastructure readiness is known as a clear and defined process to gain maximum benefit or success from any duties. But from a business point of view, infrastructure may resist IoT=based E-Learning systems due to the unfamiliarity with e-learning features, general dislike for adoption, lack of concentration, lack of sociability, lack of a qualified team, and competent people to gain profit from IoT or smart E-Learning system (Hadullo et al., 2018).

#### Online Monitoring

In online monitoring, students' action through the IoT-based E-Learning is observed to avoid the action of any prohibited activities, such as skipping the lectures, cheating, plagiarism, etc. Online monitoring is totally dependent on internet signals and speed. During COVID-19, internet traffic volume has increased by around 40%, so this caused slower download performance and raised concerns about the resilience of the Internet which directly affects online monitoring. Moreover, technical problems also influence online monitoring, such as bandwidth limitations, internet problems, and incompatible technology, which are the main reasons for the adoption of IoT in E-Learning systems (Elzainy et al., 2020).

#### Online Exams

E-Examinations or Internet-based questionnaires are referred to as online exams. The virtual examination allows students to give the exam from anywhere in the world. Lack of computer and Internet skills, fear of technology, and cheating in exams since there are no actual supervisors to invigilate and control the exam flow are the main barriers to online examination in E-Learning. However, conducting an online examination is easy because the automatic timer in online examination allows students to give the exam in the allocated time, and after this, the system automatically saves and closes the exam interface. To validate a student's identification, the e-proctor requires a fingerprint scanner, as well as an eye tracker with a camera to detect the user's eye movements while, online questionnaire design, online monitoring, automatic marking, and time setting for the online exam are easily managed by instructors in the virtual hall (Muzaffar et al., 2021).

#### Training

The act of teaching a skill or process to a person is called training. Training is essential for the instructors since there is a lack of competent computer instructors to shift from old conventional teaching techniques to smart or IoT-based E-Learning systems. The development and execution of corporate training programs customized to the demands of a specific educational institution is the most effective type of instructor training and reorientation in the smart learning environment. The lack of conducting e-learning in a disciplined and efficient way is the main reason for not having properly trained staff in HEIs (Liu et al., 2018).

#### User-Friendly

User-friendly means a system that is easy to use and understand both by students and instructors. Acceptance of technology by users is essential in developing and executing E-Learning methods in an organization. User satisfaction is affected by usability difficulties, such as information quality, interface issues, control, navigation, and flexibility issues. User satisfaction and usability are interconnected with each other. Therefore, the IoT E-Learning system user interface is easy to be understood by both students and instructors (Malik et al., 2020).

### Technological Factors

Technical devices or tools are one of the essential considerations when it comes to implementing IoT-based E-Learning. Instructors and students must know how to utilize technology, and IoT-based devices and platforms must be simple for them to access and use. IoT-based E-Learning systems should have a solid framework and have the ability to add any needed content easily and quickly. They must also have sufficient IoT resources to make effective use of it. Another point to consider is that the system should have enough network and data security so that users can rely on it. Furthermore, for each type of inquiry, the technical support system may be set up to resolve users' issues (Razzaque and Hamdan, 2020a). Technological factors are described in the following subsections.

#### Awareness

Awareness refers to having knowledge or perception regarding the adoption and implementation of IoT-based E-Learning. Students and instructors are unaware of the benefits of the adoption of IoT or smart devices in HEIs. To overcome this factor, seminars and briefings on E-Learning awareness must be incorporated as a part of the academic curriculum. For the adoption of e-learning, the educational institution must establish appropriate regulations and procedures. When users log into an online account, they should be given the required information on how to use the IoT-based E-Learning system to avoid any unethical activity (Kumar, 2018).

#### Ease of Access

By using an IoT-based E-Learning system, students can also select an instructor-guided or self-paced learning approach that provides them an opportunity to access e-learning resources on and off-campus easily. On E-Learning portals, lectures and activities are valuable and easy to access just by clicking on the link provided. Students and instructors need a very minimum of technological resources to enable E-Learning, such as the Internet and electricity (Alhabeeb and Rowley, 2018).

#### Ease of Use

Ease of use is an idea that illustrates how easily both students and instructors can use the IoT E-Learning system. Ease of use is a metric that evaluates user satisfaction. From the students' perspective, the student also chooses the ideal time to study at any place. Moreover, they can change the speed of learning to their requirements or their understanding abilities. From the instructors' perspective, the online teaching and exam, uploading course material and attachments, posting assignments, checking assignment status, and giving e-learning operating instructions related to the course are very effortless due to IoT (Nugroho et al., 2018).

#### Efficient Structure

An efficient structure is a learning system that provides maximum productivity. The number of students in virtual classrooms or E-Learning systems might be extremely low or high without affecting the overall cost. The IoT-based E-Learning has a positive impact on a learning system and increases student engagement. The IoT-based E-Learning reduces the traveling cost of students, decreases material purchasing costs, and improves the overall performance of students (Srivastava, 2019).

#### Multimedia Installment

Internet of things-based devices or learning types of equipment, such as iPad, smart boards, smart tablets, video cameras, computers, and multimedia devices are used in the domain of e-learning education systems to enhance the student's performance. The installation of a multimedia system has several benefits, including maintaining the course in front of a huge audience, recording, and preserving data (Mayer, 2017). The inclusion of video and audio resources by instructors in E-Learning makes the learning process more enjoyable, which helps students in remembering lessons for a long time.

#### Network and Data Security

Network and data security protect against breaches, invasions, and other dangers related to the network and data. This broad word (Network and data security) encompasses hardware and software procedures, regulations, settings about network use, accessibility, and overall threat prevention. Access control, application security, firewalls, network analytics, several forms of network-related security (endpoint, web-based, wireless), virus and antivirus software, VPN encryption, and more are all part of the network security. Defending client data and information, keeping shared data safe, maintaining dependable access and network performance, and protecting against cyber-attacks require network and data security. So, network and data security play an essential role in the IoT-based E-Learning in HEIs as the whole system needs to be protected from threats and risks (Kumar et al., 2020).

#### Reliability

The term reliability means to be consistent and trustworthy in terms of performance. The presence of reliable and trust connections between users and E-Learning systems encourages students to participate effectively in learning activities. Reliable technical infrastructure leads to high-course interactivity between instructors and students through an IoT-based E-Learning system. Moreover, a high level of reliability means a high level of system acceptability by users (Hashim et al., 2020).

#### System Resources

The resources of IoT-based E-Learning systems are the components that provide its inherent capabilities and contribute to its overall performance, such as IoT smart devices, networks, virtual classrooms, virtual exam halls, e-invigilator, conference rooms, course materials, etc. Computer applications are used to offer study materials that are quite simple and easy to be executed. E-Learning technologies are used to transmit knowledge and skills among students to enhance the overall performance in the HEIs (Farsakhanova, 2020).

#### Time/Support for Learning

E-Learning systems allow study materials to be updated more often than classroom-based education systems. Once the study materials are in the online platform, they will be updated without having to replace the entire set, and they can be accessed and reused for an extended period. Students can also use the system to download and save learning materials for later use that support them by providing a high-tech learning experience (Maravanyika et al., 2017).

#### Technical Support

Required software and tools are provided by the HEIs among students and instructors for the IoT-based E-Learning system. Using web-based e-learning technologies makes learning more efficient. Moreover, excessive mental effort is not required when interacting with the e-learning system, and students become more skillful and up to date in interacting with IoT e-learning technologies (Vershitskaya et al., 2020).

### Environment Factors

The learning environment is based on classrooms and homes. Furthermore, it should be preferable to teach their students and instructors before publishing the course to understand the IoT-based E-Learning system entirely. Again, several particular problems, such as privacy and ethical issues, should be considered while operating an online classroom. Moreover, the meeting or conference room, class capacity, and internet speed must be considered. The faculty should also be responsible for providing the necessary assistance and IoT resources for the class to operate the IoT-based E-Learning system correctly (Amasha et al., 2020). Environmental factors are described in the following subsections.

#### Class Capacity

IoT-based E-Learning provides the facility with unlimited class capacity. There is no specific number of participants who can join the virtual classroom. The e-link is provided to the participants for joining the class. Instructors utilize the room to address students during a virtual live lecture in real-time. There is an extra option available in an online class where the instructor can easily upload an unlimited number of course materials for the students. Students can download the course materials at any time and any place (Eze et al., 2020).

#### Internet Speed

The speed of the Internet connection is known as the bandwidth that is essential for the success of IoT-based E-Learning systems. Sometimes due to heavy traffic and load on E-Learning systems and the lack of broadband signals in particular locations might result in financial losses of HEIs and become the cause of damage to E-Learning systems (Azlan et al., 2020).

#### Legal and Ethical Issues

Legal issues are based on laws, such as data protection, copyright, and plagiarism. Ethical issues are based on the difference between right or wrong, such as academic honesty, privacy, and surveillance. There is no academic system that is completely free from plagiarism. Many instructors and education administrators are concerned that the introduction of e-learning has made it simple for students to access almost infinite written resources, putting them at risk of academic cheating. Administrators must establish a positive and supportive e-learning environment and act legally when students or instructors attempt to break legal and ethical laws (Satterfield and Kelle, 2017).

#### Meeting/Conference Room

The purpose of meeting rooms is to facilitate group discussions and conversations. IoT based-E-Learning enables students to collaborate on projects and other group assignments by using a meeting or conference room. At the same time, it facilitates instructors to prepare course designs, assignments, and projects for the students. For these purposes, these conference rooms are designed with advanced features. Instructors can submit course outcome and evolution reports to a presentation board for administration to watch and assess. Real-time feedback regarding students' projects and assignments maintains students' interest and keeps them engaged in E-Learning activities (Marky et al., 2019).

#### Privacy

The ability to keep information from becoming public is known as privacy. In IoT-based E-Learning, a private cloud is a cloud platform that is dedicated to a single instructor or student. Internally, IoT-based E-Learning is handled by instructors and students, or externally by the organization. This prominent feature prevents many security issues, but it may be costly for small institutes or universities in terms of affordability. Students' preferences, grades, assignments, learning history, and learning outcomes may be automatically recorded by the IoT-based E-Learning systems (Husain and Budiyantara, 2020).

#### Progress

Students' progress in E-Learning is known as having an idea of whether students have learned and how much they gain knowledge efficiently. Instructors who participate in IoT-based E-Learning must have a basic knowledge of the system as well as the ability to use E-Learning instruction effectively. Rather than being a passive observer of E-Learning material, each student may choose learning activities that are best suitable for individual background, interests, and a job at the time (Razzaque and Hamdan, 2020a).

#### Support of Faculty

Support of faculty is essential in reaching IoT-based E-Learning objectives, such as easy accessibility, consistency, accurate message, and smart technology. Effective, extensive education, and training are required by faculty instructors and students. To satisfy such demands, faculty must be able to create successful IoT-based E-Learning content. To assist IoT-based E-Learning, the faculty must incorporate instructional methodologies to organize new knowledge and skill workshops, such as seminars or practice tasks (Ueda and Ikeda, 2017).

#### Tools

Internet of things-based E-Learning devices and tools are used to enhance the interaction between instructors and students that becomes quite simple just by clicking the right mouse button on online class links or objects and selecting an action from a menu option. Tools are designed to assist students to learn programming by demonstrating how to utilize problem-solving strategies and programming easily and interactively. Learners will never lose patience with an E-Learning system, and electronic resources will always be of high quality and are well-maintained (Mangesa, 2021).

## Comparative Analysis of the Influencing Factors for the Adoption of IoT for E-Learning in HEIs

In this section, we perform a comparative analysis of the influencing factors for the adoption of IoT for E-Learning in HEIs based on Table 2.

**Table 2:** E-Learning influencing factors for IoT adoption in HEIs.

	**Individual factors**												**Organizational factors**								**Technological factors**										**Environmental factors**							
	**Instructors/students**												**Institutes/universities**								**Devices/tools**										**Class room/home**							
**References**	**Ability**	**Anxiety**	**Auto-attendance mechanism**	**Attitude**	**Behavior**	**Computer competency**	**Feedback**	**Interaction**	**Interactive learning**	**Motivation**	**Self-efficiency**	**Skills**	**Course design**	**Financial constraints**	**Financial readiness**	**Infrastructure readiness**	**Online exam/test/quiz**	**Online monitoring**	**Training**	**User friendly**	**Awareness**	**Ease of access**	**Ease of use**	**Efficient structure**	**Multimedia installation**	**Network and data security**	**Reliability**	**System resources**	**Time/support for learning**	**Technical support**	**Class capacity**	**Internet speed**	**Legal and ethical issues**	**Meeting/conference room**	**Privacy**	**Progress**	**Support of faculty**	**Tools**
Sezer et al. (2017)								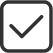		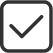																									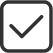			
Zhamanov et al. (2017)						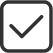	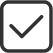	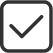	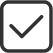	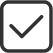		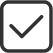							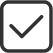		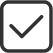	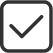	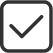						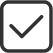									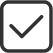
Lei et al. (2017)								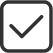	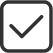	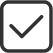	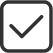	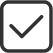																										
Qasem et al. (2019)		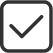		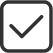	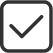	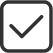		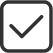	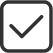		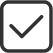			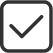		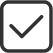						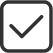	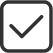			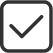	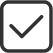	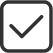							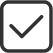			
Popchev et al. (2019)																								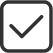				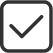		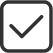								
Kamarudin et al. (2019)										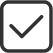	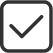							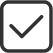								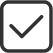									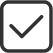			
Wangoo and Reddy (2020)									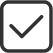									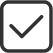	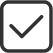									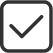									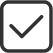	
Ejaz et al. (2020)						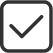																										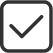						
Rico-Bautista et al. (2019)																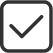					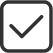			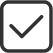		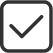	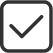						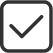		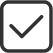		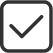	
Kaizer et al. (2020)											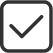								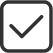		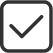								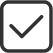	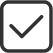							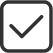	
Chweya and Ibrahim (2021)				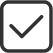												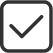		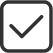								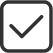									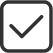			
Saini and Goel (2019)				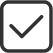				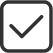								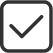									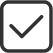						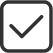			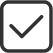				
Gul et al. (2017)														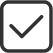										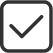		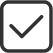				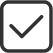		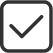			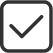			
Fernández-Caramés and Fraga-Lamas (2019)																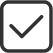	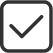									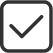			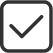						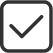			
Priatna et al. (2020)	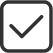			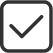								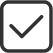				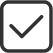						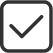	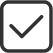				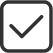											
Gunesekera et al. (2019)				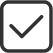				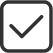										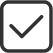				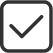	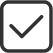							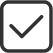								
Zahedi and Dehghan (2019)										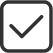				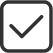		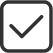			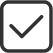							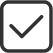									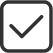			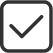
Pervez et al. (2018)						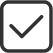	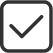	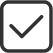	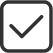		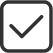			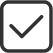														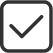	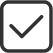					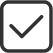				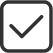
Khan and Salah (2020)									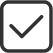															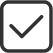		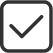		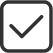					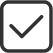		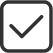			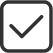
Leong and Letchumanan (2019)													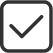	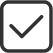						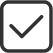																		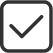
Muzaffar et al. (2021)														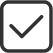		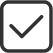	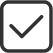	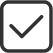	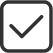				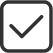			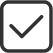												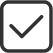
Letting and Mwikya (2020)			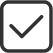											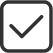		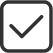										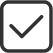	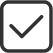								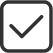			
Almazroi et al. (2016)		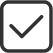		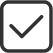																			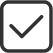				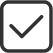											
Ngampornchai and Adams (2016)				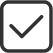							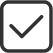		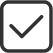				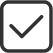				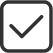			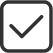														
Kanwal and Rehman (2017)		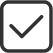				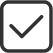					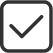											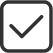	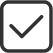							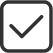								
Jović et al. (2017)																						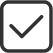	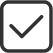	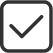														
El-Masri and Tarhini (2017)				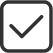						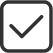				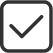	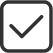								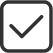				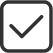											
Naveed et al. (2017)		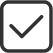		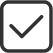		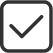		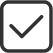		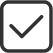	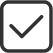	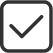	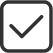		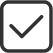	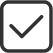			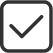		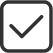	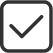	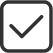	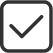		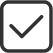	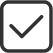			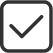		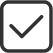	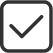					
Naveed et al. (2019)													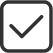	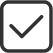									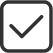				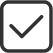			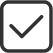			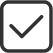				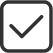	
Alhabeeb and Rowley (2018)	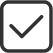																		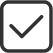			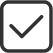	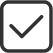					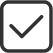		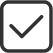							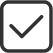	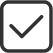
Hamidi and Chavoshi (2018)					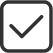																		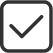				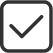											
Rahardjo (2018)					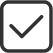					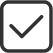		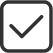						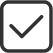				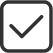								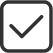		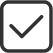					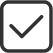	
Muhammad et al. (2020)								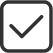										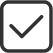												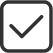								
Al-Araibi et al. (2019b)												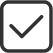			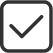	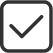				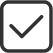		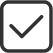	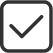	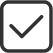		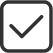				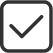		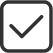						
Eze et al. (2018)				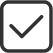	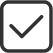			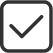	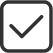													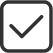	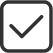															
Salloum et al. (2019)					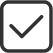			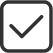	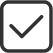														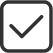	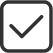			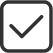										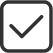	
Al-Araibi et al. (2019a)																						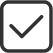	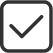			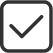		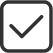		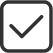								
Hasani et al. (2020)								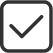		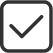	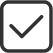	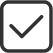		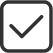	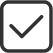	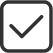						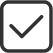				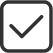			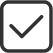	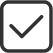		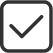						
Amasha et al. (2020)								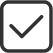	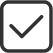					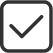														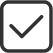		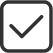					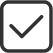			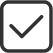
Mehta et al. (2019)	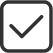				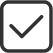					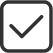	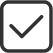	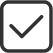											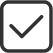															
Kim et al. (2019)				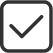				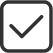	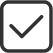							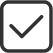																				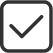		
Pérez et al. (2021)	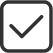			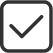					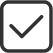		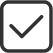																				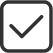							
Shaikh H. et al. (2019)					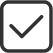									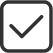									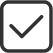			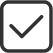	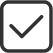	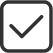							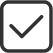	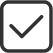		
Shinghal et al. (2020)						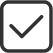													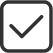	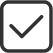			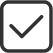					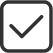										
Kuliya and Usman (2021)				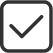	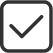									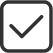								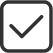	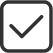						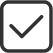								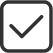	

**Figure 3:**
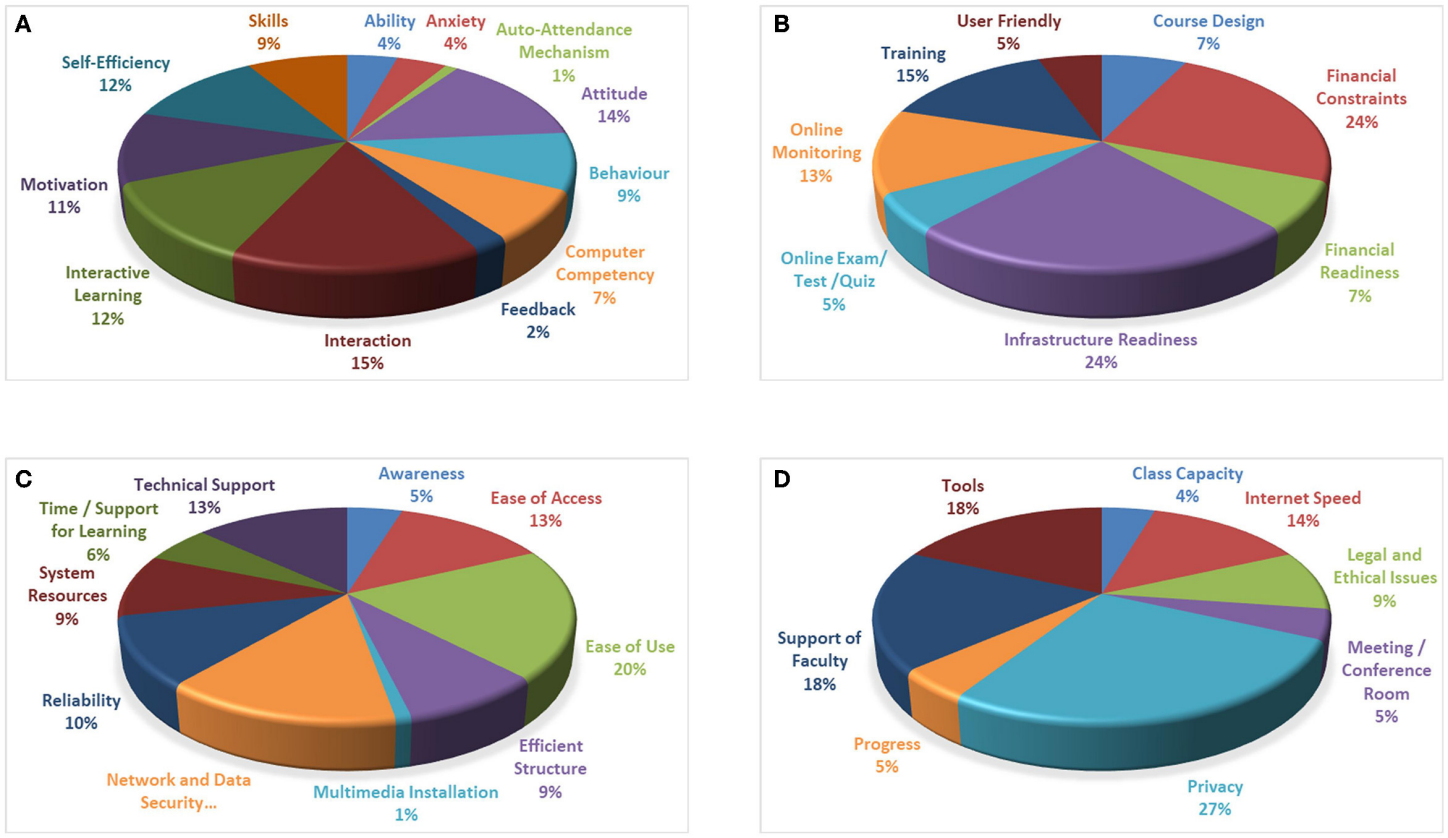
Percentage of E-Learning influencing factors for IoT adoption in HEIs. **(A)** Individual factors. **(B)** Organizational factors. **(C)** Technological factors. **(D)** Environmental factors.

Figure 3A highlights the individual influencing factors of IoT adoption for E-Learning in HEIs, based on the instructors and students. Interaction (15%) relates to the participation of instructors and students in any activities of class participation. The IoT-based E-Learning allows users to connect at any time, allowing them to address their problems; that is why interaction has taken the highest significant factor. If the instructor and teacher have a good attitude (14%) toward E-Learning, positive outcomes will arise; dire consequences may result if they have a negative attitude toward the adoption of IoT-based E-Learning. Next, this evaluation will be directed to interactive learning and self-efficiency, which stand for 12% of the total each. Self-efficiency relates to a user's belief in their ability to do a specific job efficiently and effectively. On the other hand, interactive learning refers to self-regulation of E-Learning through video viewing or group activities. Later, motivation, skills, behaviors, and computer competency have covered an excellent space as the influencing factors for the adoption of IoT-based E-Learning in HEIs, while the auto-attendance mechanism (1%), feedback (2%), ability (4%), and anxiety (4%) are received less consideration by the researchers for the adoption of IoT-based E-Learning in HEIs. Even though these factors have important values for the adoption of IoT-based E-Learning, they also affect the performance of the students in their results. Feedback refers to a person's thoughts or ideas for improvement, and the interaction technique can also be used to provide feedback, which may be the reason why it received less consideration than the auto-attendance mechanism. However, at the end of everything, the auto-attendance mechanism, feedback, ability, and anxiety also need to focus along with interaction, attitude, interactive learning, self-efficiency, and motivation for a better outcome for using IoT-based E-Learning in HEIs.

Similarly, Figure 3B shows the organizational (institutions or universities) influencing factors that are needed to be a concern for IoT adoption in E-Learning. Infrastructure readiness (24%) means to gain the maximum benefits from any option for the adoption of the IoT-based E-Learning that has got the highest consideration, so it proves researchers' hope to get the maximum benefits from the latest IoT-based E-Learning mechanism. On the other side, financial constraints (24%) refer to the cost of developing and maintaining the high-tech required for IoT-based E-Learning. Again, based on the figure, training (15%) secures the second consideration as it is necessary to train the instructor with the tech to make sure they can teach properly with the help of IoT-based E-Learning. Training is also required for the students for learning the usage of IoT-based E-Learning devices, tools, and platforms. Then, online monitoring, financial readiness, and course design received average consideration by the researchers. Moreover, Online Exam/Test/Quiz (5%) and user-friendly interfaces (5%) have reached the lowest percentage in the organizational influencing factors. So researchers need to be more focused on Online Exam/Test/Quiz and user-friendly interfaces along with infrastructure readiness, financial constraints, training, and online monitoring for the adoption of IoT-based E-Learning in HEIs.

In the same way, Figure 3C emphasizes the technological influencing factors. It shows that ease of use (20%) has been claimed as the top consideration by the researchers. Ease of use guarantees that users can quickly use the IoT-based E-Learning system, including logging in, signing out, and other user-friendly interfaces. Network and data security (14%) also takes a significant consideration, which is essential to protect client data and information, preserve shared data, ensure dependable access, and network performance, and protect against cyber-attacks. Then comes technical support (13%) and ease of access (13%), tied for a more considerable influence. Technical support refers to the provision of necessary software and tools for the proper operation of IoT-based E-Learning among students and instructors. Ease of access (13%) is also a vital influencing factor for IoT-based E-Learning since it allows users to access it anywhere at any time. Reliability and system resources have also received a higher consideration by the researchers. In contrast, multimedia installation (1%), awareness (5%), and time and support for learning (6%) have received relatively low values as compared to the other influencing factors, with multimedia installation receiving the most negligible value. Multimedia installation is an important influencing factor, so it requires more consideration. As a result of the overall comparison, multimedia installation, awareness, and time and support for learning need more consideration from the researchers, along with the influencing factors like ease of use, network security, and data security are the essential technological elements for the adoption of IoT-based E-Learning in HEIs.

Lastly, Figure 3D illustrates the environmental factors (classroom or home), which shows that the influencing factor privacy is way far prioritized from other factors, with the highest consideration of 27% by the researchers. Privacy is the ability to keep information secure and seems to be one of the most important ones on the list of environmental factors. Next, tools (18%) and the support of faculty (18%) have jointly taken the second priority for the IoT-based E-Learning by the researchers. For continuing the IoT-based E-Learning, tools are needed to utilize appropriately. In the case of faculty support, it is essential for easy accessibility, consistency, and proper technical support. The influencing factors of internet speed (14%) and legal and ethical issues (9%) have also got reasonable consideration. Still, class capacity (4%), meeting conference room (5%), and progress (5%) have got less consideration whereas class capacity has got the lowest consideration. So, from the figure, it is clear that environmental factors, class capacity, meeting conference room, and progress need more concern along with privacy, tools, support of faculty, and the Internet for the adoption of IoT-based E-Learning in HEIs.

Hence, researchers need to be more focused on these influencing factors depending on individuals, organizational, technological, and environmental for the adoption of IoT-based E-Learning in HEIs.

## Conclusion and Recommendations

The Internet of things has become a significant technology in ensuring to attainment of the needs of educators and educated learners. This helps in improving the quality of education in developing countries. Even though Corona ruled the globe, IoT aided in continuing the educational system. Students can study from home with the aid of IoT, and the Internet has provided a plethora of resources to help them learn effectively. Understanding the key factors that influence IoT may help the Ministry of Higher Education and university decision-makers plan their strategies. The research study examines the influencing factors that are significant for the IoT adoption of E-Learning in HLIs in developing countries by designing an E-Learning-based IoT adoption Model. These influencing factors are categorized into individual, organizational, environmental, and technological groups. The comparative analysis of these influencing factors is presented in a detailed description in order to determine which factors should be prioritized for efficient IoT-based E-Learning in HEIs. The findings will be helpful for university policymakers and the government to address the factors and make well-informed decisions regarding IoT adoption for E-Learning. It will help provide support and encourage teaching staff and students to incorporate these technologies into their learning. The result will be high quality educational outcomes.

## Data Availability Statement

The original contributions presented in the study are included in the article/supplementary material, further inquiries can be directed to the corresponding author.

## Author Contributions

SHHM and JA: conceptualization. SHHM and JS: data curation. SHHM and SM: formal analysis, investigation, and software. SHHM: methodology and visualization. JA and SH: project administration and supervision. SM, JS, and MM: resources. JA and JS: validation. SHHM, HH, and MHM: writing–original draft and writing–review and editing. All authors contributed to the article and approved the submitted version.

## Funding

The authors are thankful to Saudi Electronic University (SEU) and Universiti Teknologi Malaysia (UTM) for supporting this research through RUG grant No. IFRS 7873.

## Conflict of Interest

The authors declare that the research was conducted in the absence of any commercial or financial relationships that could be construed as a potential conflict of interest.

## Publisher's Note

All claims expressed in this article are solely those of the authors and do not necessarily represent those of their affiliated organizations, or those of the publisher, the editors and the reviewers. Any product that may be evaluated in this article, or claim that may be made by its manufacturer, is not guaranteed or endorsed by the publisher.
